# Selective Enhancement of Photoresponse with Ferroelectric‐Controlled BP/In_2_Se_3_ vdW Heterojunction

**DOI:** 10.1002/advs.202205813

**Published:** 2023-02-13

**Authors:** Jian Wang, Changlong Liu, Libo Zhang, Jin Chen, Jian Chen, Feilong Yu, Zengyue Zhao, Weiwei Tang, Xin Li, Shi Zhang, Guanhai Li, Lin Wang, Ya Cheng, Xiaoshuang Chen

**Affiliations:** ^1^ State Key Laboratory of Precision Spectroscopy East China Normal University Shanghai 200062 P. R. China; ^2^ State Key Laboratory of Infrared Physics Shanghai Institute of Technical Physics Chinese Academy of Sciences 500 Yu Tian Road Shanghai 200083 P. R. China; ^3^ Hangzhou Institute for Advanced Study University of Chinese Academy of Sciences No.1 SubLane Xiangshan Hangzhou 310024 P. R. China; ^4^ Shanghai Research Center for Quantum Sciences 99 Xiupu Road Shanghai 201315 P. R. China

**Keywords:** depletion region, ferroelectric polarization, photoresponse enhancement, vdW heterojunction, *α*‐In_2_Se_3_

## Abstract

Owing to the large built‐in field for efficient charge separation, heterostructures facilitate the simultaneous realization of a low dark current and high photocurrent. The lack of an efficient approach to engineer the depletion region formed across the interfaces of heterojunctions owing to doping differences hinders the realization of high‐performance van der Waals (vdW) photodetectors. This study proposes a ferroelectric‐controlling van der Waals photodetector with vertically stacked two‐dimensional (2D) black phosphorus (BP)/indium selenide (In_2_Se_3_) to realize high‐sensitivity photodetection. The depletion region can be reconstructed by tuning the polarization states generated from the ferroelectric In_2_Se_3_ layers. Further, the energy bands at the heterojunction interfaces can be aligned and flexibly engineered using ferroelectric field control. Fast response, self‐driven photodetection, and three‐orders‐of‐magnitude detection improvements are achieved in the switchable visible or near‐infrared operation bands. The results of the study are expected to aid in improving the photodetection performance of vdW optoelectronic devices.

## Graphical abstract

A ferroelectric‐enabled van der Waals (vdWs) heterojunction through vertically stacking *α*‐In2Se3 and black phosphorus (BP) is reported. The energy bands at the heterojunction interfaces can be aligned and flexibly engineered. Fast‐response, self‐driven photodetection, and three‐orders of magnitude detection improvement are achieved in the switchable visible or near‐infrared bands. This work provides a feasible method for the performance improvement of vdWs optoelectronic devices.

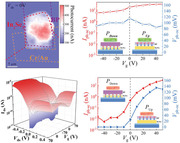

## 1 Introduction

As core components, heterojunction‐based light‐emitting or photodetection devices are crucial to underpinning modern information science and technology. Compared with their bulky counterparts, two‐dimensional (2D) materials provide an outstanding platform for the construction of high‐performance electronic/optoelectronic structures in an ultracompact manner, particularly, for infrared photodetectors. It facilitates the random stacking of different 2D materials to form various functional heterojunctions without lattice mismatch; however, this severely restricts the performance of traditional covalent or ion‐bonded devices.^[^ ^1^, ^2^, ^3^, ^4^, ^5^ ^]^ In addition, the asymmetric built‐in electric field at the heterojunction interface enables dark current suppression and a self‐driven photoresponse with a high signal‐to‐noise ratio.^[^ ^6^ ^]^ Pioneering studies on van der Waals heterojunction photodetectors have been reported. Lee et al. reported gate‐tunable photovoltaic (PV) responses with atomically thin heterodiodes based on the stacking structures of n‐MoS_2_ and p‐WSe_2_.^[^ ^7^ ^]^ Ghosh et al. presented a high‐speed WSe_2_/SnSe_2_ diode with large negative responsivity through band alignment engineering.^[^ ^8^ ^]^ Remarkably, Wang et al. demonstrated the first observation of a ballistic avalanche in vertical InSe/BP heterostructures.^[^ ^9^ ^]^ Moreover, several other vertically stacked heterojunctions, such as BP/MoS_2_,^[^ ^10^ ^]^ GaTe/MoS_2_,^[^ ^11^ ^]^ and AsP/InSe^[^ ^12^ ^]^ have been explored. The underlying mechanism relies on the transportation of photogenerated carriers excited in intrinsic channels or depleted p‐n regions. However, the precise control and tuning of doping concentrations remain a common challenge in 2D materials, hindering the photodetection performance of such PV detectors.^[^ ^13^, ^14^, ^15^ ^]^ It is worth noting that electrostatic doping can significantly relieve this problem; however, it is achieved at the cost of a dramatic decrease in photoabsorption and quantum efficiency because it reduces the thickness of the photoactive area to avoid the electrostatic shielding effect.

The advent of 2D ferroelectric materials has ushered a versatile pathway for realizing versatile doping control. Compared with bulky ferroelectric films, which impose high demands on the growth substrates to reduce the lattice mismatch and manufacturing process to avoid defects,^[^ ^16^ ^]^ 2D ferroelectric layers allow high‐quality interfaces, a better combination of nanostructures, and a higher integration density. Indium selenide (In_2_Se_3_), a representative ferroelectric III‐VI compound with a monolayer comprising alternating Se or In atoms through covalent bonding, has demonstrated great potential in electronics and optoelectronics.^[^ ^16^, ^17^ ^]^ Notably, *α*‐phase n‐In_2_Se_3_ exhibits a stable structure at room temperature, and its out‐of‐plane polarization^[^ ^18^, ^19^ ^]^ can be efficiently engineered via the application of an external electric field at different temperatures.^[^ ^20^, ^21^, ^22^ ^]^ Thus, combined with other 2D materials to construct different heterojunctions, 2D ferroelectric materials are expected to yield more flexible

This study proposed a ferroelectric‐enabled van der Waals heterojunction by vertically stacking *α*‐In_2_Se_3_ and BP to realize high‐sensitivity photodetection in switchable visible and infrared bands. By tuning the ferroelectric polarization field, the carrier concentration at the interface was controlled to selectively deplete the carrier concentration in the In_2_Se_3_ or BP layers. Consequently, the depletion region was broadened and shifted. Moreover, switchable high‐performance detection was achieved in both the visible and infrared wavelengths by flipping the applied direction of the ferroelectric polarization field. The proposed strategy promises a feasible solution for overcoming the tradeoff between light harvesting and wavelength selection in conventional photodetectors.

## 2 Results

### 2.1 BP/In_2_Se_3_ vdW Heterojunction Fabrication

**Figure 1:**
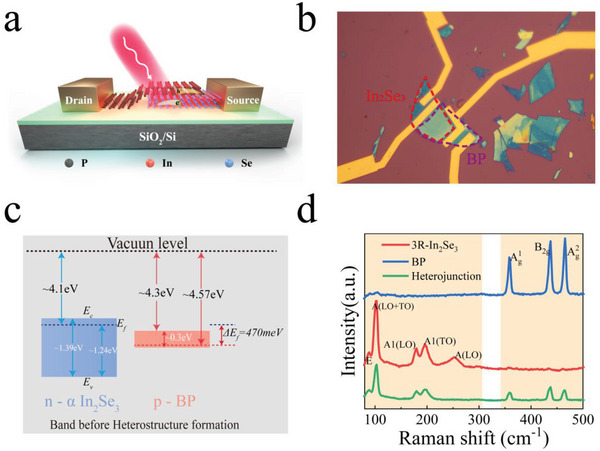
Schematic of the heterojunction device and the characterizations on the band alignment and Raman spectra. a) Schematic illustration of the gated black phosphorus (BP)/indium selenide (In_2_Se_3_) van der Waals (vdW) heterojunction. A SiO_2_ layer with 280 nm thickness is used as the bottom dielectric on the top of heavily doped Si. b) Optical microscope image of the device with top source/drain contacts on individual BP and In_2_Se_3_ layers. Purple and red marked areas represent BP and In_2_Se_3_ flakes, respectively. c) Energy band diagrams of BP and In_2_Se_3_ flakes before stacking into heterojunction. d) Raman spectra of individual BP and In_2_Se_3_ flakes, as well as the overlapping heterojunction area.

A schematic of the BP/In_2_Se_3_ vdW heterojunction is shown in **Figure** **1**a. The device was placed on a heavily p‐doped Si/SiO_2_ substrate, and BP layers were placed above the In_2_Se_3_ material. This configuration facilitated better light absorption in the BP and easy implementation of the gate voltage from the substrate. A thin film of the photoresist PMMA was spin‐coated above the device to isolate the air‐induced degradation after preparation. The position of the middle Se atom changes under a perpendicular electric field induced by the gate voltage. This breaks the spatial symmetry and affects the ferroelectric polarization state.^[^ ^23^ ^]^ An optical micrograph of the device is shown in Figure 1b. To eliminate the effect of various resistances, electrodes were manufactured near the boundaries of the overlapping heterojunction, similar to previous reports.^[^ ^24^ ^]^ No air bubbles existed in the overlapping region of the heterojunction, except for a few impurities on the surface, indicating that the heterojunction had excellent interfacial quality. The line‐scanning profiles of the heterostructures obtained via atomic force microscopy (AFM) are shown in Figure S1 (Supporting Information). The thicknesses of the BP and In_2_Se_3_ flakes were ≈10 nm. Owing to the absence of in‐plane polarization in 3R *α*‐In_2_Se_3_ for this thickness,^[^ ^25^ ^]^ the effect of in‐plane polarization was not observed in the following measurement.

To characterize the energy‐band alignment between 3R *α*‐In_2_Se_3_ and BP, ultraviolet photoelectron spectroscopy (UPS) measurements were performed, as shown in Figure S2 (Supporting Information). Both the valence band spectrum and second electron cutoff energy (*E*_cut_) of In_2_Se_3_ were measured. The work function (*W*) of In_2_Se_3_ was estimated to be 4.1 eV and the Fermi level was 1.24 eV above the valence band according to formula *W* = *hv* – *E*_cut_, where *hv* = 21.2 eV is the photon energy of the He source. To accurately obtain the bandgap of 3R *α*‐In_2_Se_3_, the photoluminescence spectra of the bulk crystals were also measured from 1.2 to 1.6 eV. With Lorentz fitting, a direct bandgap of ≈1.38 eV was achieved (see Figure S3, Supporting Information), which is consistent with the results of previous studies.^[^ ^26^, ^27^ ^]^ The energy band details of BP were extracted.^[^ ^15^ ^]^ The equilibrium energy band diagrams of the individual BP and In_2_Se_3_ flakes are shown in Figure 1c. The Raman peaks of BP, 3R *α*‐In_2_Se_3_, and their overlapping positions are shown in Figure 1d. For BP, three distinct peaks corresponding to $A_{g}^{2}$, B_2g_, and $A_{g}^{1}$ vibrational modes were observed.^[^ ^28^ ^]^ Whereas, for 3R *α*‐In_2_Se_3_, together with *E*‐symmetric and A(LO + TO) modes, the A1(LO) and A1(TO) modes were observed owing to symmetry breaking of the atom structure (belonging to the R3m space group).^[^ ^29^, ^30^ ^]^ This provides direct evidence that 3R *α*‐In_2_Se_3_ is different from its *β* phase counterparts, which are stable at room temperature.^[^ ^31^ ^]^ In a previous study, the presence of the A(LO) mode indicated a lack of antisymmetry in the R3m structure.^[^ ^30^ ^]^ In particular, the observation of A(LO) mode further confirmed the ferroelectric 3R *α*‐In_2_Se_3_ crystal structure.^[^ ^34^ ^]^ Compared with the Raman spectra of individual materials, there was no dramatic decrease in the peak intensities and significant translation of the peak positions at the heterojunction. This implies high crystallinity in the overlapping area. Moreover, the disappearance of A(LO) peak of *α*‐In_2_Se_3_ can be attributed to the absorption of BP, which restricts the incident light from reaching the *α*‐In_2_Se_3_ material.

### 2.2 Characterizations on Dark Current of the BP/ In_2_Se_3_ Heterojunction

First, the electrical characteristics of BP and In_2_Se_3_ FETs were measured in a dark environment. The linear *I*–*V* and Ohmic behaviors at different gate voltages (see Figure S4a,c, Supporting Information) indicate a reduction in the barrier height for carrier transport in BP and In_2_Se_3_.^[^ ^32^ ^]^ In addition, the transferring curves measured at biases of 0.05 and 0.2 V confirmed the ambipolar‐behavior of BP device with on/off ratio over 10^3^. Moreover, the transfer curve of the In_2_Se_3_ device exhibited clockwise ferroelectric hysteresis with a current on/off ratio of over 10^5^. Consequently, a conduction state at a positive gate voltage (+*V*_G_) occurred. In contrast to other ferroelectric dielectrics with only polarization‐bound charges, the ferroelectric semiconductor (3R *α*‐In_2_Se_3_) hosts both mobile and ferroelectric‐bound charges, benefiting from the cooperative behavior of the semiconductor and ferroelectric properties.^[^ ^33^ ^]^ Further, details can be found in Figure S4b,d (Supporting Information).

**Figure 2:**
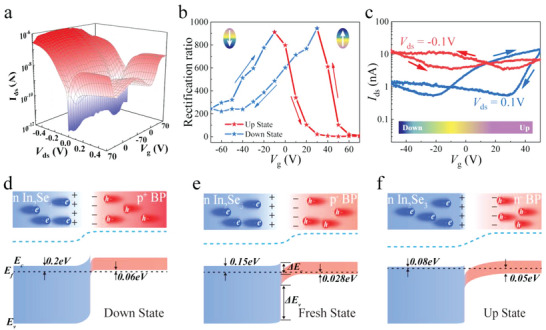
Electrical characterizations of the black phosphorus (BP)/indium selenide (In_2_Se_3_) van der Waals (vdW) heterojunction. a) Three‐dimensional (3D) surface plot of *I*_DS_ for the BP/In_2_Se_3_ vdW heterojunction as functions of *V*_DS_ and *V*g. b) Illustration of the “Up” and “Down” states. It implies the gate‐dependent hysteresis rectification ratio of the BP/In_2_Se_3_ vdW heterojunction. c) Transferring characteristics of the vdW heterojunction under forward voltage *V*_ds_ = 0.1 V and reverse bias voltage *V*_ds_ = −0.1 V, respectively. d–f) Schematic demonstration of the energy band structure for BP/In_2_Se_3_ vdW heterojunction at “Down,” “Fresh,” and “Up” states, respectively.

For the stacked In_2_Se_3_/BP device, the BP (drain) was grounded to avoid the influence of the applied voltage on the out‐of‐plane polarization in In_2_Se_3_. The output current curves for the different ferroelectric polarization states are shown in **Figure** **2**a. The rectification ratio (*I*_DS0.5 V_ / *I*_DS‐0.5 V_) curves in Figure 2b reveal a clear ferroelectric hysteresis with a tuning range of up to three orders of magnitude. The similar hysteresis loops can be attributed to the water molecules, oxide charge trapping, and surface charge trapping functioning as adsorbents.^[^ ^34^, ^35^, ^36^ ^]^ Thus, to reduce the influence of these uncertainties, the measurement was performed in a vacuum environment at 1.8 × 10^−5^ mbar. Furthermore, a 180° change in the piezoresponse phase (see Figure S7, Supporting Information) was observed owing to the reversal of the out‐of‐plane (OOP) polarization of In_2_Se_3_ under an external electric field. The phase hysteresis loop exhibited two opposite remnant polarization states. The coercive electric field was estimated to be ≈300 kV cm^−1^, which is of the same order of magnitude as the gate electric field needed in Figure 2b for tuning the hysteresis of the rectification ratio. This further verifies the indispensable role of ferroelectric polarization field. The phase hysteresis loop was asymmetric with respect to the bias axis (see Figure S7, Supporting Information), which can be attributed to the difference in the work function between the AFM tip and *α*‐In_2_Se_3_.^[^ ^18^, ^37^ ^]^ Moreover, because a residual ferroelectric polarization field exists in *α*‐In_2_Se_3_, the peak of the rectification ratio was not located near zero gate voltage. This results in the threshold behavior of the ferroelectric polarization field.

The rectification curve shown in Figure 2b can be divided into two parts. Polarization field in In_2_Se_3_ operates in the “Down” state among the gate voltage *V*_G_ sweeping from 30 to −70 and −70 to −10 V. In this case, BP exhibited heavily P^+^ doping and In_2_Se_3_ was weakly n‐doped. The depletion region was mainly located on the In_2_Se_3_ side. For the “Up” state of gate voltage *V*_G_ changing from 70 to 30 V and −10 to 70 V, BP exhibits light n‐type doping and In_2_Se_3_ is n^+^‐type, which results in the depletion of the BP side. Therefore, the transfer characteristics are primarily dependent on the carrier concentration, which was controlled via the ferroelectric polarization field at the heterostructure interface. The ferroelectric hysteresis was observed in the transfer curves under 0.1 and −0.1 V drain bias as shown in Figure 2c, which further validates the effect of ferroelectric polarization field.

The equilibrium band alignments corresponding to the three different polarization states are shown in Figure 2d–f. According to Figure 1c, the conduction and valence band differences were determined as ▵ *E*_c_= 0.35 eV and ▵ *E*_v_= 0.74 eV. In addition, using the transfer curves in Note S1 (Supporting Information), the field‐effect mobility of BP was obtained as 201 cm^2^V^−1^s^−1^ and a bulk‐carrier concentration of 1.48 × 10^18^ cm^−3^ in the heterojunction. Similarly, a carrier concentration of 2.42 × 10^16^ cm^−3^ in 3R *α*‐In_2_Se_3_ was obtained from the Hall test (details in Figure S6, Supporting Information). At this stage, (*E*_f_ − *E*_v_) for p‐doped BP and (*E*_c_ − *E*_f_) for n‐doped In_2_Se_3_ were determined as 0.028 and 0.14 eV (see Note S1, Supporting Information), respectively. The value of (*E*_c_ − *E*_f_) for In_2_Se_3_ was consistent with the UPS measurements. Further, the Fermi‐level difference ▵*E*_f_ of 0.47 eV was calculated for these two materials before stacking. Because the interfacial state of the heterojunction can be neglected owing to the presence of the dangling bond‐free structure, the distribution of the depletion region was mainly determined by the doping concentrations of the two materials. From the band diagrams, the dark current in the “Up” state at zero bias is lower than in the other states because the narrow bandgap BP is depleted, which substantially reduces the dark current generated by thermal excitation, which is consistent with the test results in Figure 2a. To obtain precise distributions at different polarization states, performed theoretical calculations (see Note S2, Supporting Information) were performed to determine the widths of the depletion region across the interface. From the above results, it can be concluded that the depletion region distribution of the BP/In_2_Se_3_ vdW heterojunction can be selectively controlled through the effective modulation of carriers at the interface by the ferroelectric field, which promises significant enhancement of the photoresponse.

### 2.3 Photoresponse Performance of the BP/In_2_Se_3_ vdW Heterojunction

**Figure 3:**
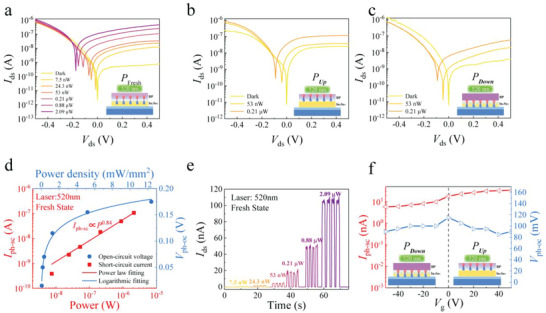
Photoresponse performance of black phosphorus (BP)/indium selenide (In_2_Se_3_) van der Waals (vdW) heterojunction in the visible region. *I*_DS_–*V*_DS_ curves at a) “Fresh” state, b) “Up” state, and c) “Down” state under different incident powers. d) Power‐dependent short‐circuit photocurrent *I*_ph‐sc_ and open‐circuit photovoltage *V*_ph‐oc_. e) Time‐resolved photocurrent in the “Fresh” state with 520 nm incident light with different powers. f) Gate voltage dependent short‐circuit photocurrent *I*_ph‐sc_ and open‐circuit voltage *V*_ph‐oc_ at a fixed optical power of ≈0.21 µW.

To characterize the photodetection performance of the BP/In_2_Se_3_ vdW heterojunction, photoresponse measurements were performed in the visible and near‐infrared bands. **Figure** **3**a–c shows the *I*_DS_–*V*_DS_ characteristics at different light intensities of 520 nm light irradiation for three ferroelectric polarization states‐ “Fresh,” “Down,” and “Up,” respectively. Figure 3a shows the distinct PV response. The photogenerated carriers imposed a continuous shift in *I*_DS_–*V*_DS_ curve at different incident powers, resulting in a short‐circuit photocurrent (*I*_ph‐sc_) and open‐circuit voltage (*V*_ph‐oc_). As shown in Figure 3d, the *I*_ph‐sc_ curve exhibited an approximately linear increase with optical power (by power function fitting *I*_ph‐sc_ ∝ *P* ^0.84^). However, *V*_ph‐oc_ exhibited a logarithmic relationship with optical power (see Figure S8, Supporting Information, and Figure 3). Optical switching curves for different optical powers are shown in Figure 3e. The ratio of zero‐bias photocurrent to dark current at “Fresh” state reached 1 × 10^4^, representing a high signal‐to‐noise contrast. Further, the PV responses of the heterojunction in different ferroelectric polarization states were also measured. The relationship between the open‐circuit voltage and short‐circuit current, along with the change in the ferroelectric polarization state at a wavelength of 520 nm, is shown in Figure 3f. For “Up” state, short‐circuit current increased slightly with more efficient charge separation in the depletion region. However, for “Down” state, the depleted region was primarily occupied by In_2_Se_3_ and the diffusion of minorities carriers was inhibited at the heavily doped BP region. The increase under the “Up” state can be attributed to the widening of the depletion region in BP as it would contribute to the 520 nm wavelength photoresponse (see Note S2, Supporting Information). The ferroelectric hysteresis curves under illumination are shown in Figure S9c,d (Supporting Information).

**Figure 4:**
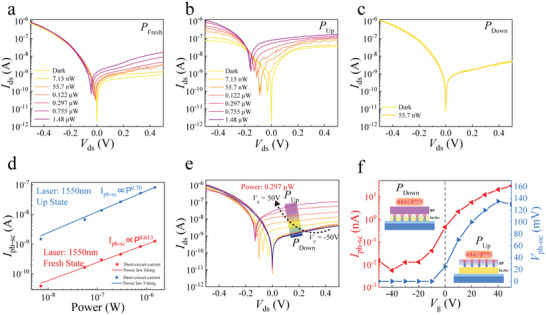
Photoresponse performance of black phosphorus (BP)/indium selenide (In_2_Se_3_) van der Waals (vdW) heterojunction in the near‐infrared region. Photocurrents of the heterojunction at different incident powers for ferroelectric polarization field in a) “Fresh” state, b) “Up” state, and c) “Down” state. d) Power‐dependent short‐circuit photocurrent *I*_ph‐sc_. e) *I*_ds_–*V*_ds_ curves for different gate voltages applied to the heterojunction. Incident optical power is fixed at 0.297 µW. f) Gate voltage dependent short‐circuit photocurrent *I*_ph‐sc_ and open‐circuit voltage *V*_ph‐oc_ at different polarization states.

In_2_Se_3_ has a bandgap of ≈1.38 eV and cannot satisfy the requirements of conventional applications such as transceivers, modulators, and detectors^[^ ^24^, ^38^, ^39^ ^]^ operating in the near‐infrared region. However, the integration of BP into the proposed vdW heterojunction allows for a significant PV response at 1550 nm, as shown in the *I–V* curves in **Figure** **4**a. It is evident that when the ferroelectric polarization direction of In_2_Se_3_ was changed by the gate‐voltage, the “Up” state exhibited a significant enhancement of the PV response while the “Down” state yielded a minimal response to incident light. This is in contrast to the case of visible illumination. To investigate the enhancement of the ferroelectric polarization state on the NIR photoresponse, we measured the power‐dependent photoresponse in the “Up” state, as shown in Figure 4b. The *I*_DS_ *–V*_DS_ curve shifted upward with increase the optical power. This increase was more significant than that shown in Figure 4a. The short‐circuit photocurrents *I*_ph‐sc_ in the “Fresh” and “Up” states both increased in a linear relationship with optical power (Fresh: *I*_ph‐sc_ ∝ *P* ^0.70^; Up: *I*_ph‐sc_ ∝ *P* ^0.613^) as shown in Figure 4d. However, the open‐circuit photovoltage *V*_ph‐oc_ was logarithmically related to the optical power (see Figure S10, Supporting Information). The different behavior of the open‐circuit photovoltage *V*_ph‐oc_ suggests that interface doping resulted in a shift in the Fermi level owing to the ferroelectric polarization field. Thus, to evaluate the PV response enhancement of the ferroelectric‐controlled BP/In_2_Se_3_ vdW heterojunction, the short‐circuit photocurrent *I*_ph‐sc_ and open‐circuit photovoltage *V*_ph‐oc_ were obtained when the In_2_Se_3_ polarization states changed from “Down” state to “Up” state under 0.297 µW optical power in Figure 4e,f. The short‐circuit photocurrent *I*_ph‐sc_ changed by three orders of magnitude as the ferroelectric polarization state flips. This can be primarily attributed to the widening of the depletion region in BP (see Note S2, Supporting Information), which is consistent with the band alignment discussion under ferroelectric control in Figure 2. At this time, the photogenerated carriers generated in BP are rapidly absorbed by the metal electrode under the action of the built‐in electric field. Following this operation mechanism, it can be concluded that short‐circuit current *I*_ph‐sc_ increases when tuning the ferroelectric polarization from “Down” state to “Up” state in both Figures 3f and 4f.

However, the PV voltage *V*_ph‐oc_ is determined by the resistance and photocurrent. The resistance that decreases a bit under “Up” polarization is determined by the ferroelectric polarization according to the band alignment. The photocurrent at 1550 nm in Figure 4f increased more rapidly than that at 520 nm (Figure 3f) when the ferroelectric polarization was tuned. This phenomenon can also be explained by the band alignment because photocarriers were more efficiently generated in the BP region at a longer wavelength, and the quantum efficiency was improved significantly by the depletion of BP. Moreover, the photocarriers generated in the In_2_Se_3_ region at 520 nm in Figure 3f exhibited a wider bandgap than that of BP. This improved the photon absorption at 520 nm, whereas the photocurrent change was less significant than that at 1550 nm by changing the ferroelectric polarization. Similarly, the transfer curves at a fixed bias voltage also exhibited a significant enhancement in the photovoltage response in the “Up” state. Further details can be found in Figure S11c,d (Supporting Information).

**Figure 5:**
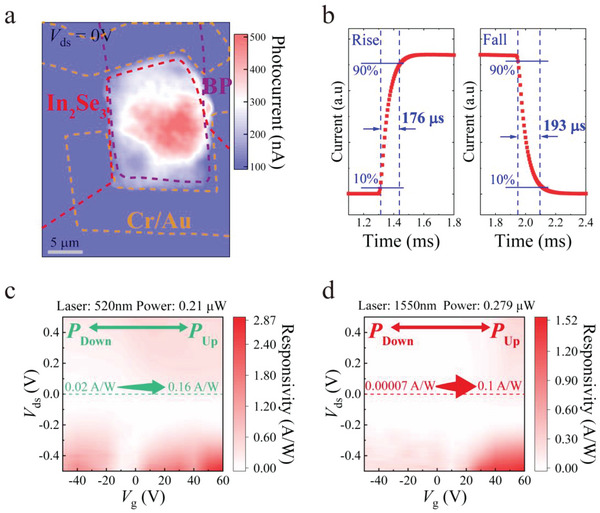
Photocurrent mapping and response time of black phosphorus (BP)/indium selenide (In_2_Se_3_) van der Waals (vdW) heterojunction. a) Scanning photocurrent mapping of the BP/In_2_Se_3_ vdW heterojunction under 520 nm laser illumination with the laser spot size less than 1 µm without external bias. b) The self‐driven time‐resolved photoresponse of the BP/In_2_Se_3_ vdW heterojunction. Responsivity mapping through scanning bias voltage *V*_ds_ and gate voltage *V*_g_ at fixed optical powers of c) 0.21 µW (520 nm) and d) 0.297 µW (1550 nm), respectively.

The primary contribution of photogenerated carriers is light absorption in the intrinsically doped or depletion regions. To evaluate the influence of different ferroelectric polarization states on the carrier concentration variation in the depletion region, the photoresponse of the BP/In_2_Se_3_ vdW heterojunction was characterized from the visible to near‐infrared region. The BP layer exhibited a photoresponse only at 1550 nm, whereas both BP and In_2_Se_3_ flakes exhibited a PV response at a wavelength of 520 nm. To confirm that the PV response mainly originated from the heterojunction rather than from the Schottky junction effect, scanning photocurrent mapping was conducted to identify the distribution of photocurrents. As shown in **Figure** **5**a, a significant photocurrent was observed throughout the junction region, whereas no obvious photocurrent was generated around the metal contact positions. Another important parameter of photodetectors is the response time. As shown in Figure 5b, the device yielded rise and fall times of 176 and 193 µs, respectively, which were up to three orders of magnitude faster than those of other In_2_Se_3_‐based photodetectors. A comparison with reported works can be found in Table S1 (Supporting Information). Compared with photogating effects, which may enhance the photoresponses in dedicatedly designed heterogeneous structures, prevalent for similar structures on SiO_2_ substrates. Despite photogating effect can contribute to a large optical response,^[^ ^40^, ^41^, ^42^ ^]^ it suffers from fatal drawbacks of slow response time. It is always in the level of tens or hundreds of milliseconds, or even seconds for the photodetectors due to the restriction of interface defects capturing effect. For photogating effect based on In_2_Se_3_, the response time is 9 s at 520 nm.^[^ ^43^ ^]^ For BP‐based device, the response time is 5 × 10^−3^ s.^[^ ^44^ ^]^ However, for In_2_Se_3_/BP heterojunction in our manuscript, the photoresponse time is in the order of microseconds for all ferroelectric polarization states (see Figure 12, Supporting Information). Besides, photogating effect features with a nonlinear or exponential relationship between the generated photocurrent and incident optical power.^[^ ^45^ ^]^ However, the photocurrent and optical power under different ferroelectric polarization states show a linear relationship in our work. The results further confirm that the photoresponse of our device mainly originated from the heterojunction PV effect, photogating effect is not the dominating. The significant improvement in response speed can be attributed to the high mobility of BP and the efficient charge separation enabled by the large built‐in field. The dynamic variation of the photoresponse versus the gate/drain voltage at a fixed optical power at 520 and 1550 nm are shown in Figure 5c,d, respectively. The photoresponsivity was calculated using the formula *R* =*I*_ph_ /*P*. As evident, the responsivity was enhanced by an order of magnitude at a wavelength of 520 nm and by three orders of magnitude at 1550 nm under a zero‐bias voltage. With reverse bias applied to the external electrode, the peak potential barrier is covered by a steep band structure and the electrons are transported more smoothly. As a result, the photocurrent is also enhanced at a sufficiently large bias. In addition, the selective photoresponse controlled by the ferroelectric polarization state promises dual‐color detection in the visible and near‐infrared regions, thereby providing a viable route for target identification in complex environments.

The optoelectronic properties of our devices are a combination of ferroelectric properties and heterojunctions. The ferroelectricity behavior of *α*‐phase indium selenide (*α*‐In_2_Se_3_) is modulated by the external electric field. It plays a role of “field transfer” effect in controlling the BP doping concentration. For “Down” state, In_2_Se_3_ is in a low doping concentration. Under the ferroelectric field, BP exhibits a heavy p‐type doping. The depletion region mainly exists in In_2_Se_3_. The induced ferroelectric field inside In_2_Se_3_ has a direction from In_2_Se_3_ to the BP material under external bias, which is the same as the built‐in field of the heterojunction. It further enhances the depletion of free carriers in In_2_Se_3_ material. The selective photogenerated current at 520 nm incidence mainly occurs in the depleted In_2_Se_3_ since its wide bandgap ≈1.39 eV is higher than that of 1550 nm photons. The generated photocarriers can be efficiently extracted by the internal built‐up field within a finite lifetime, whereas, for “Up” state, further widens the depletion width in BP material. The theoretical calculation of depletion region proves the widening of the BP depletion (see Note S2, Supporting Information, for details). The measured photoresponse at both wavelengths of 520 and 1550 nm in Figures 3f and 4f confirms that the photocurrent generation position locates in the BP depletion area. Note that, the ferroelectric polarization field driven by the gate voltage allows the BP depletion region to shift or even disappear, resulting in 1550 nm photoresponse from nothing to something. Corresponding to the band structure, the potential barrier can affect photogenerated electron transport and not affect hole transport in BP. The detection photocurrent in the “Up” state mainly comes from the hole current in BP. In this work, the vdW heterojunction by combining wide bandgap In_2_Se_3_ with ferroelectricity and narrow bandgap BP is proposed to achieve wavelength‐selective photoresponse (520 and 1550 nm) under three states controlled by external bias. Particularly, the depletion region in the BP is gradually widened to enhance the photoresponse at 1550 nm by combining the ferroelectric polarization field. In summary, the heterojunction provides a versatile platform for controlling the depletion region positions through applying different external bias. The presence of tunable ferroelectric polarization field in In_2_Se_3_ changes the doping concentration and depletion region distribution at the heterojunction interface, playing an indispensable role in affecting the energy band and thus determining the dark current and photoresponse in different states. The tunable ferroelectric field and the built‐in field both contribute the multifunctional detection properties of our device, including the wavelength‐selective behavior, low dark current, and fast response time.

## 3 Conclusions

This study proposed a van der Waals heterojunction with a ferroelectric‐controlled depletion region. The outstanding merits of heterojunctions in switchable response in the visible and near‐infrared, fast response, and large responsivity enhancement were thoroughly demonstrated by assembling the ferroelectric properties of n‐type In_2_Se_3_ and narrow‐bandgap p‐type BP materials. Remarkably, an increase of up to three orders of magnitude in the photoresponse was achieved by significantly increasing the distribution of the depletion region in BP under the ferroelectric polarized “Up” state. Further, the ferroelectric vdW heterojunction provided an approach to overcome the low photoresponse owing to the narrow and uncontrollable depletion region in previous 2D heterojunction‐based photodetectors. It is expected that the ferroelectric‐controlled BP/In_2_Se_3_ vdW heterojunctions can be employed to engineer high‐sensitivity, multifunctional, and selective fascinating detectors to satisfy various applications in different areas.

## 4 Experimental Section

### Device Fabrication

The BP/In_2_Se_3_ vdW heterojunction in this study was fabricated using the dry transfer method with polydimethylsiloxane (PDMS) carriers. The 2D In_2_Se_3_ and BP flakes were mechanically exfoliated from the bulk material provided by HQ Graphene before being transferred to highly P‐doped silicon substrates (with thermally grown 280 nm SiO_2_) and precisely aligned to overlap with each other to form heterostructures. The transfer was conducted in a N_2_‐protection glovebox to reduce the oxidation of BP. Further, electron beam lithography (EBL, FEI F50 SEM with an NPGS system) was used to define the electrode pattern. Gold metal and chromium films with thicknesses of 15 and 45 nm, respectively, were deposited via thermal evaporation as contact electrodes, followed by a standard peeling process. Before performing the measurements, a PMMA film was spin‐coated to prevent BP exposure to air and water.

### Characterizations and Measurements

Electronic properties were determined using a commercial Keithley 4200A‐SCS probe station. Response time data were measured using a Tektronix MDO3014 oscilloscope. The Raman spectra were characterized using a Lab Ram HR800 with a 514 nm excitation laser. AFM (Bruker Dimension Icon) was used to determine the thickness of the 2D materials. A UPS (PHI5000 VersaProbe III [Scanning ESCA Microprobe) Spherical Analyzer (SCA)] was used to acquire the band structures (valence band and work function) of the In_2_Se_3_ materials.

## Conflict of Interest

The authors declare no conflict of interest.

## Supporting information

Supporting Information

## Data Availability

The data that support the findings of this study are available from the corresponding author upon reasonable request.
