# Supplementary material for: Selective Enhancement of Photoresponse with Ferroelectric‐Controlled BP/In2Se3 vdW Heterojunction
Source: Adv Sci (Weinh). 2023 Feb 13;10(11):2205813. doi: 10.1002/advs.202205813 (PMC10104633; doi:10.1002/advs.202205813)
Supplement: Supplementary file 1 — Supporting Information [file ADVS-10-2205813-s001.pdf]

ADVANCED SCIENCE
Open Access

Supporting Information

for *Adv. Sci.*, DOI 10.1002/advs.202205813

Selective Enhancement of Photoresponse with Ferroelectric-Controlled BP/In_2Se_3 vdW Heterojunction

Jian Wang, Changlong Liu, Libo Zhang, Jin Chen, Jian Chen, Feilong Yu, Zengyue Zhao, Weiwei Tang, Xin Li, Shi Zhang, Guanhai Li*, Lin Wang*, Ya Cheng* and Xiaoshuang Chen**

Supplementary Materials for

Selective Enhancement of Photoresponse with Ferroelectric-controlled BP/In2Se3 vdW Heterojunction

Jian Wang, Changlong Liu, Libo Zhang, Jin Chen, Jian Chen, Feilong Yu, Zengyue Zhao, Weiwei Tang, Xin Li, Shi Zhang, Guanhai Li*, Lin Wang*, Ya Cheng*, Xiaoshuang Chen**

J. Wang, Y. Cheng

State Key Laboratory of Precision Spectroscopy, East China Normal University, 200062, Shanghai China.

E-mail: ghli0120@mail.sitp.ac.cn, clliu@ucas.ac.cn, wanglin@mail.sitp.ac.cn, ya.cheng@siom.ac.cn, xschen@mail.sitp.ac.cn

J. Wang, J. Chen, J. Chen, F. Yu, Z. Zhao, X. Li, G. Li, L. Wang, X. Chen

State Key Laboratory of Infrared Physics, Shanghai Institute of Technical Physics, Chinese Academy of Sciences, 500 Yu Tian Road, 200083, Shanghai, China.

C. Liu, L. Zhang, W. Tang, S Zhang, G. Li, X. Chen

Hangzhou Institute for Advanced Study, University of Chinese Academy of Sciences, No.1 SubLane Xiangshan, 310024, Hangzhou, China.

X. Li, G. Li, X. Chen

Shanghai Research Center for Quantum Sciences, 99 Xiupu Road, Shanghai, 201315, China

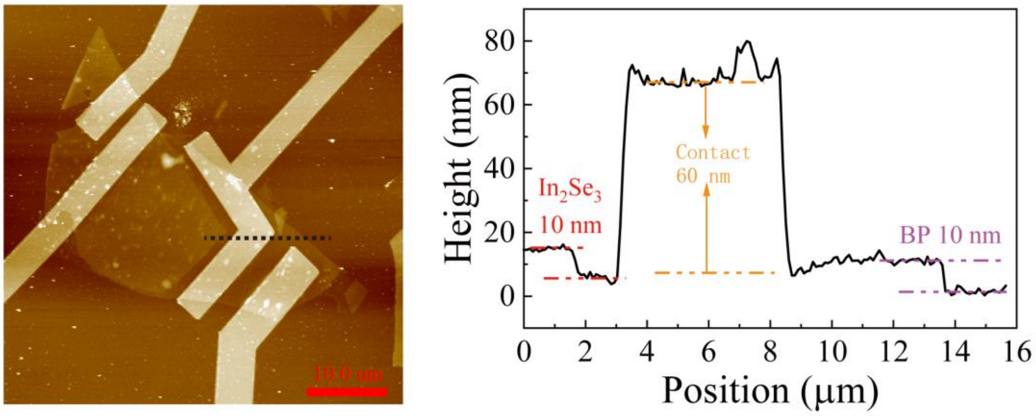

Supplementary Figure 1. AFM height measurement of the BP/In_2Se_3 vdW heterojunction device.

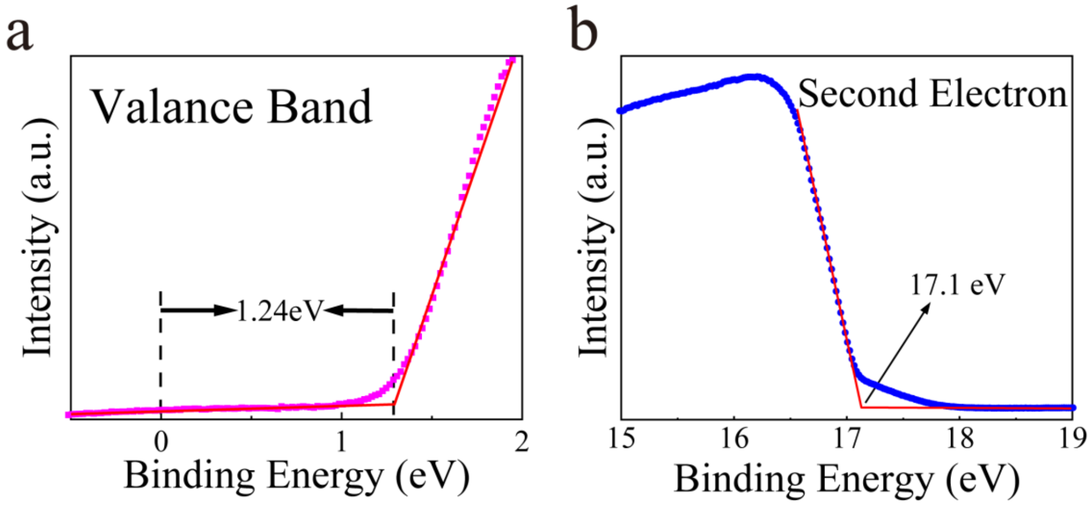

Supplementary Figure 2. UPS spectra measurement of In_2Se_3. (a) Valence band spectrum as function of binding energy. (b) Second electron cutoff point. UPS spectra were used to determine the valence band and the work function of In_2Se_3. The work function of In_2Se_3 was finally estimated to be 4.1 eV according to $W = hv - E_{cut}$, where hv = 21.2 eV is the photon energy of the He I source. It' s Fermi level locates around 1.24 eV which is above the valence band of In_2Se_3.

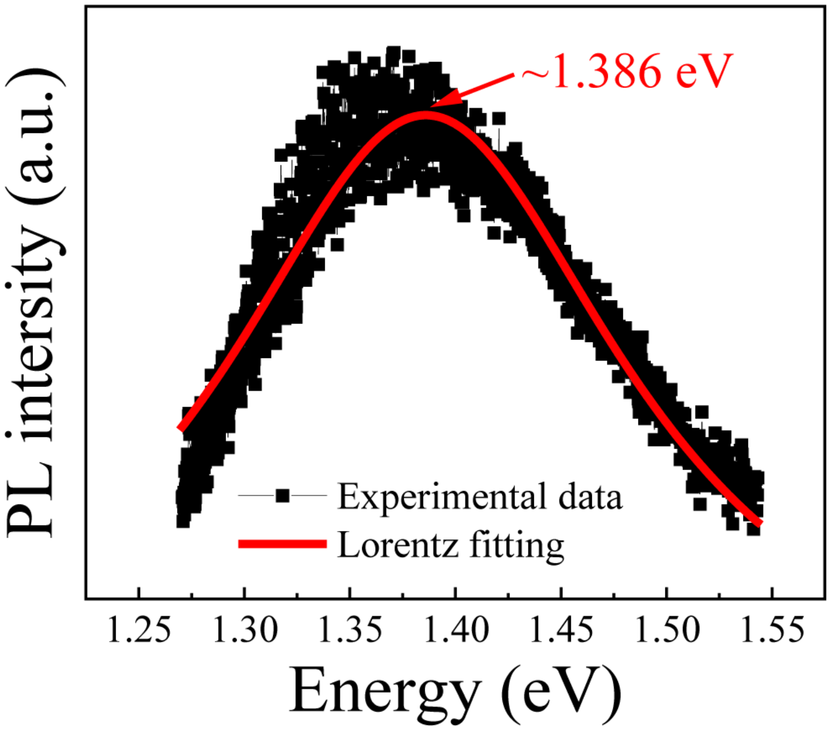

Supplementary Figure 3. Photoluminescence (PL) spectrum of In_2Se_3. The bandgap can thus be determined around 1.39 eV.

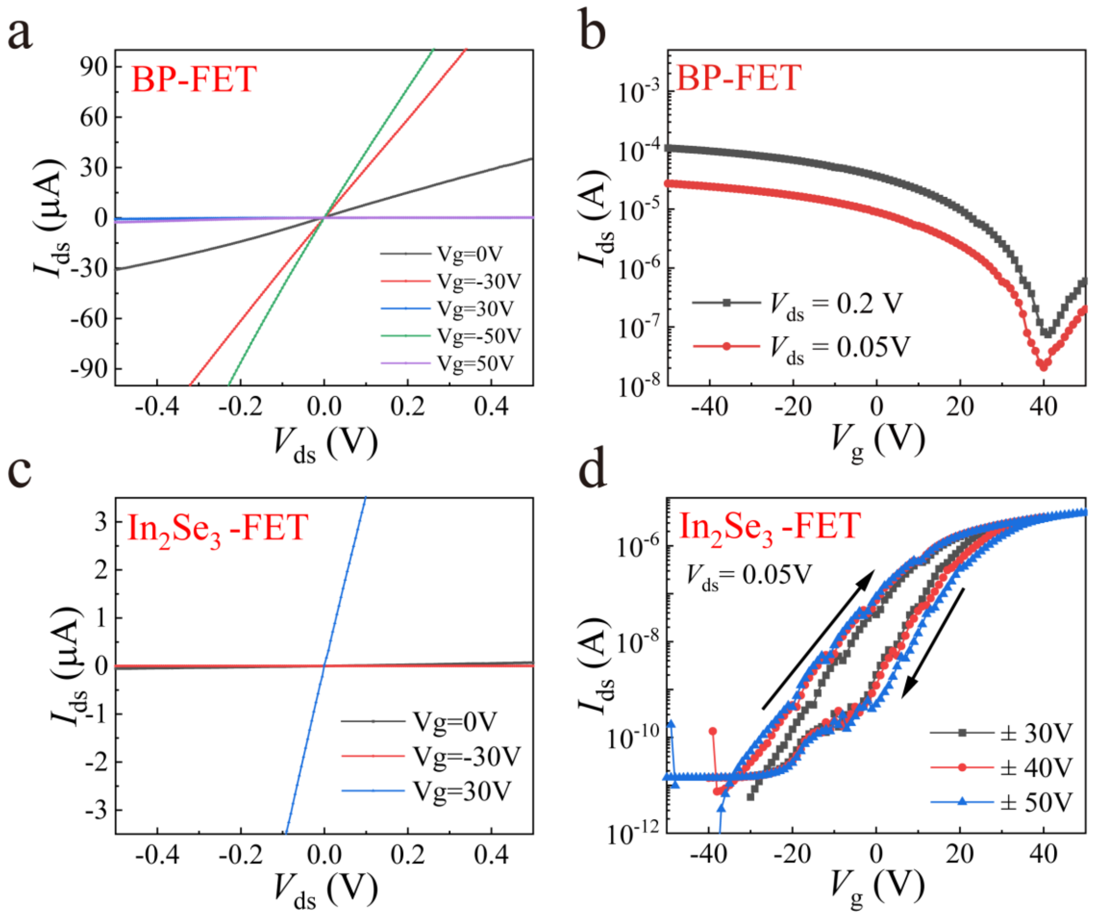

Supplementary Figure 4. I_{ds}-V_{ds} and I_{ds}-V_g measurements of individual BP and In_2Se_3 FETs. (a) Output I_{ds}-V_d curve and (b) Transfer characteristics I_{ds}-V_g of the BP transistor with Cr/Au electrodes, demonstrating p-type device characteristic with ohmic contact. (c) Output curve I_{ds}-V_d and (d) transfer characteristics I_{ds}-V_g of the In_2Se_3 transistor with Cr/Au electrodes. It confirms the n-type characteristic with ohmic contact and gate voltage-dependent ferroelectric hysteresis characteristics.

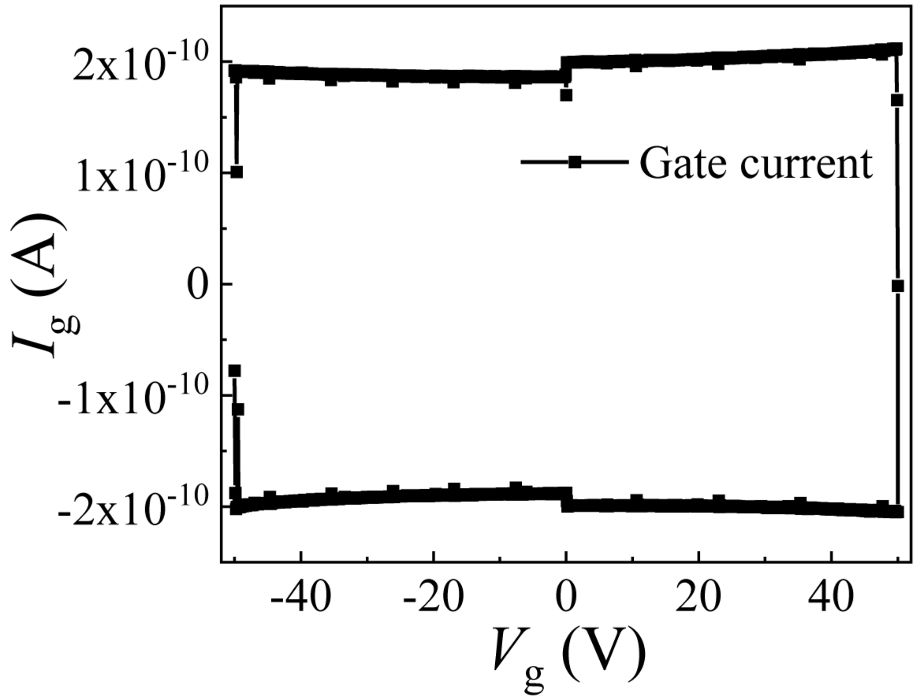

Supplementary Figure 5. Gate voltage current is monitored when measuring the transfer curve through applying back gate bias. It indicates that the device is not affected by the leakage voltage.

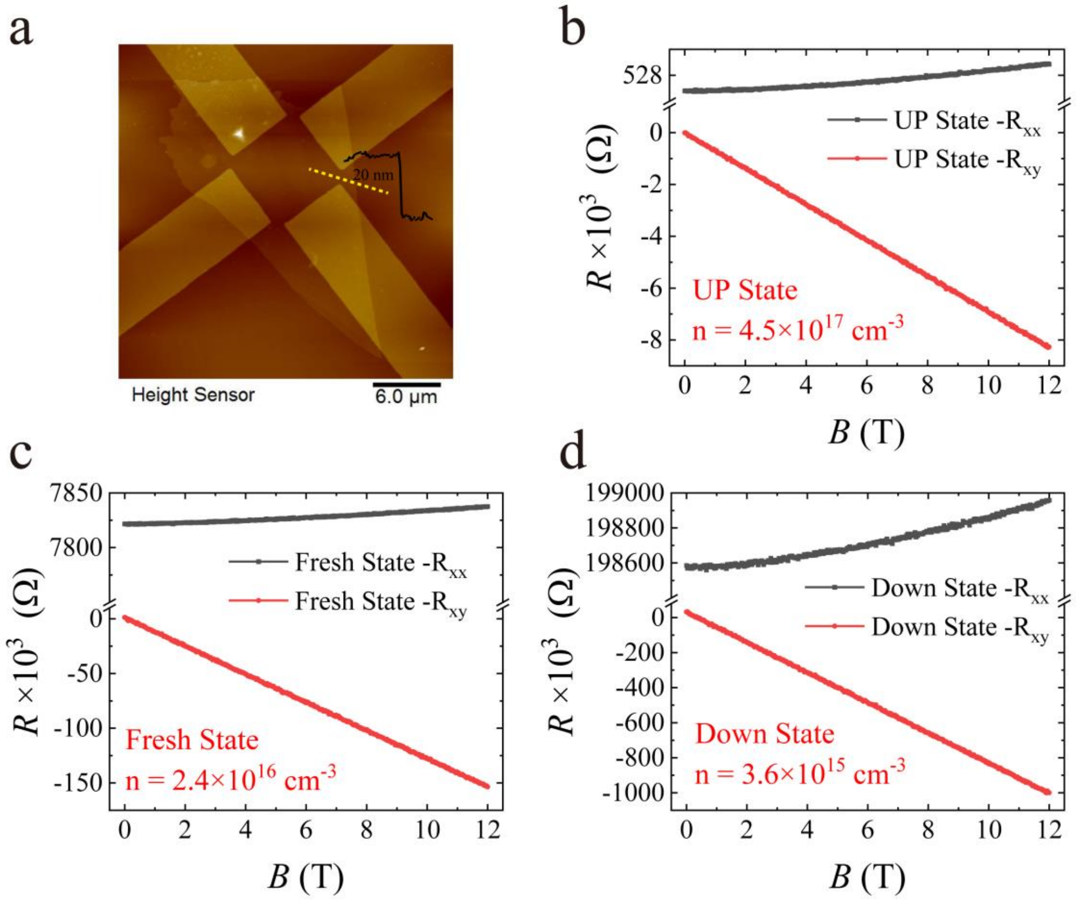

Supplementary Figure 6. Resistance-magnetic field characteristics of a α-In_2Se_3 FET with 280 nm SiO_2 as gate dielectric at room temperature. The device has channel length 10 μm, width 10 μm and thickness of 20 nm. (a) AFM image of In_2Se_3 device for Hall test and thickness characterization. R_{xy}-B characterization at (b) "Fresh" state, (c) "Up" state and (d) "Down" state. The measured electron volume concentrations are 2.42×10^{16} cm^{-3}, 4.5×10^{17} cm^{-3} and 3.6×10^{15} cm^{-3}, respective.

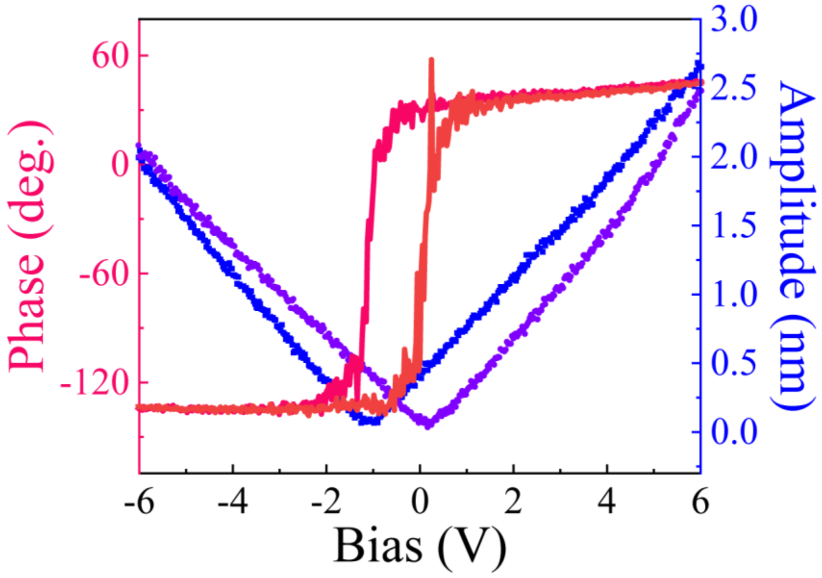

Supplementary Figure 7. Polarization reversal measurement under external electrical field. The on-field PFM phase and amplitude hysteresis loops are obtained with a 22 nm-thick flake.

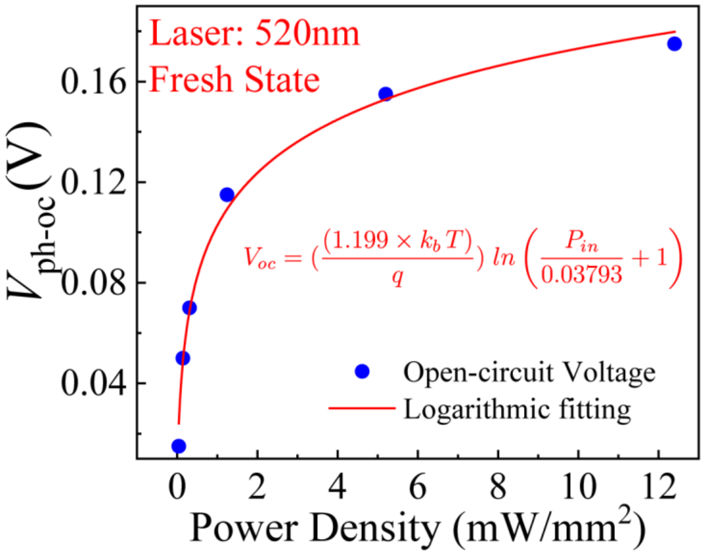

Supplementary Figure 8. Power dependent open-circuit photovoltage V_{ph}-$_{oc}$ as function of incident power. Solid symbol is the experimental data and solid curve is the logarithmic fitting under 520 nm illumination. In_2Se_3 here is in the "Free" state.

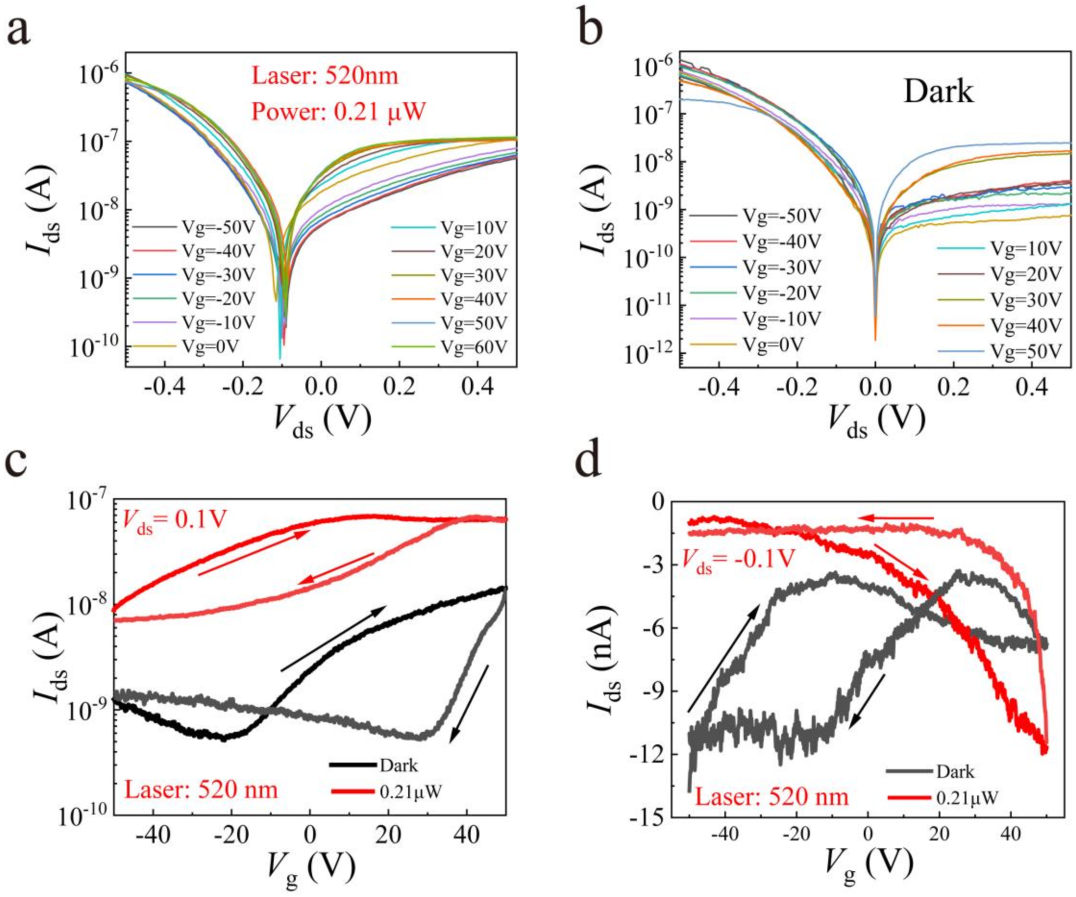

Supplementary Figure 9. Photoresponse performance of BP/In_2Se_3 vdW heterojunction in visible. (a) Output characteristics under different gate voltages at a fixed optical power 0.21 μW. (b) Output characteristics under different gate voltages at dark environment. Transfer characteristics of the vdW heterojunction at (c) forward bias voltage 0.1 V and (d) reverse bias voltage -0.1 V with/without optical power irradiation.

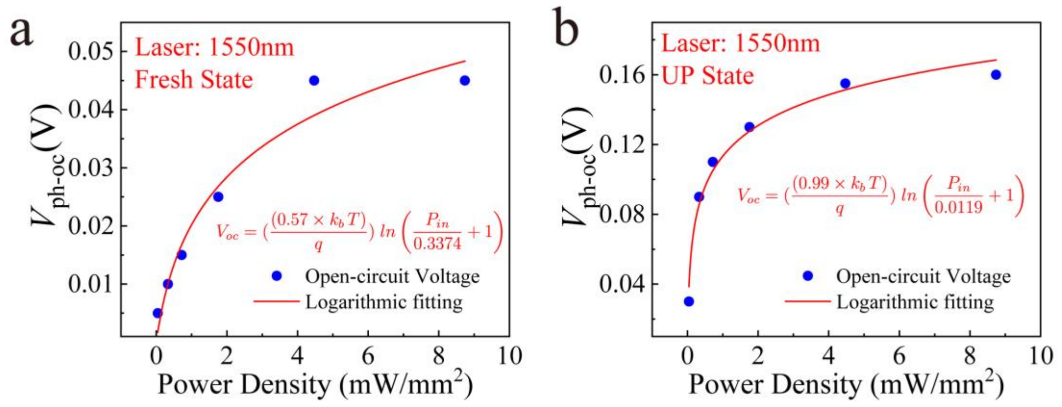

Supplementary Figure 10. Power dependent open-circuit voltage photovoltage $V_{ph\text{-}oc}$ under (a) 520 nm illumination, "Fresh" state and (b) 1550nm illumination, "Up" state. Solid symbol is the experimental data and solid curve is the logarithmic fitting.

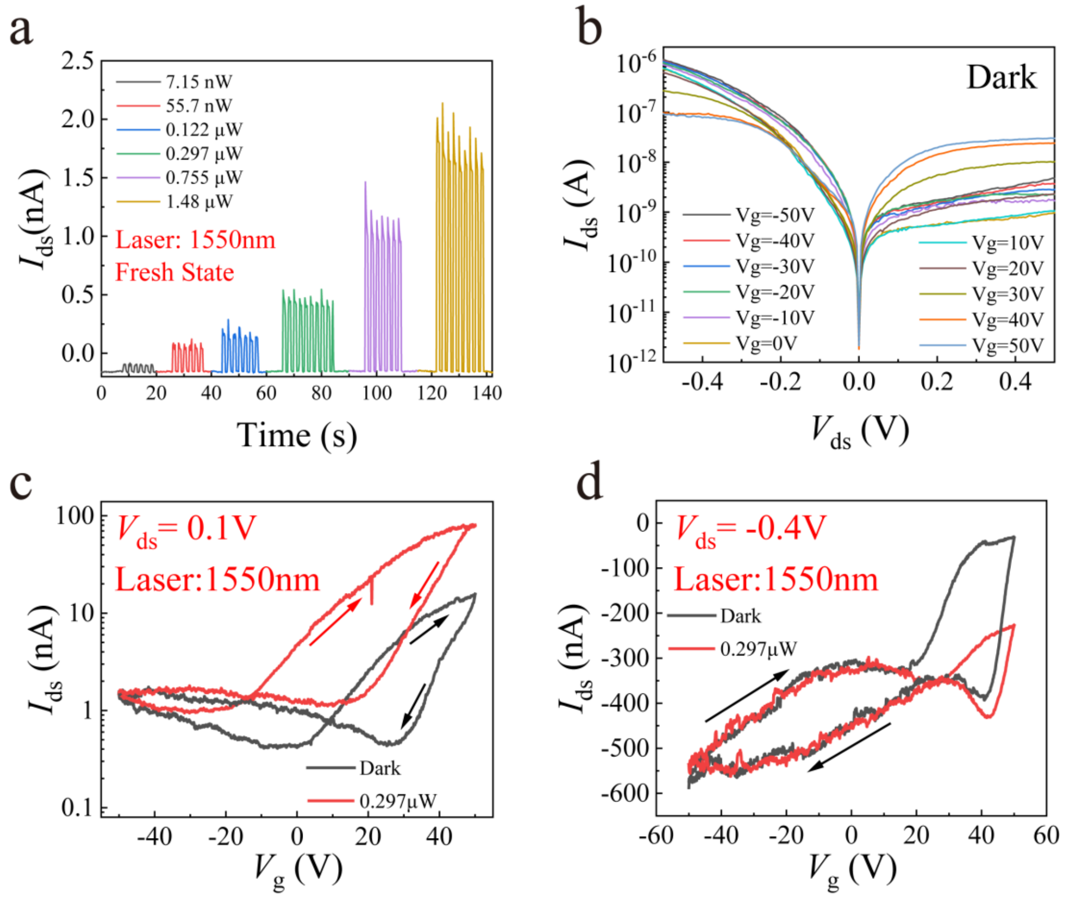

Supplementary Figure 11. Photoresponse performance of BP/In_2Se_3 vdW heterojunction in near-infrared. (a) Time-resolved photocurrent in the "Fresh" State with 1550 nm incident light and different light powers. (b) Output characteristics under different gate voltages at dark environment. Transfer characteristics of the vdW heterojunction under (c) Reverse bias voltage of 0.1 V and (d) Forward bias voltage of -0.4 V under 0.297 μW with/without optical power irradiation.

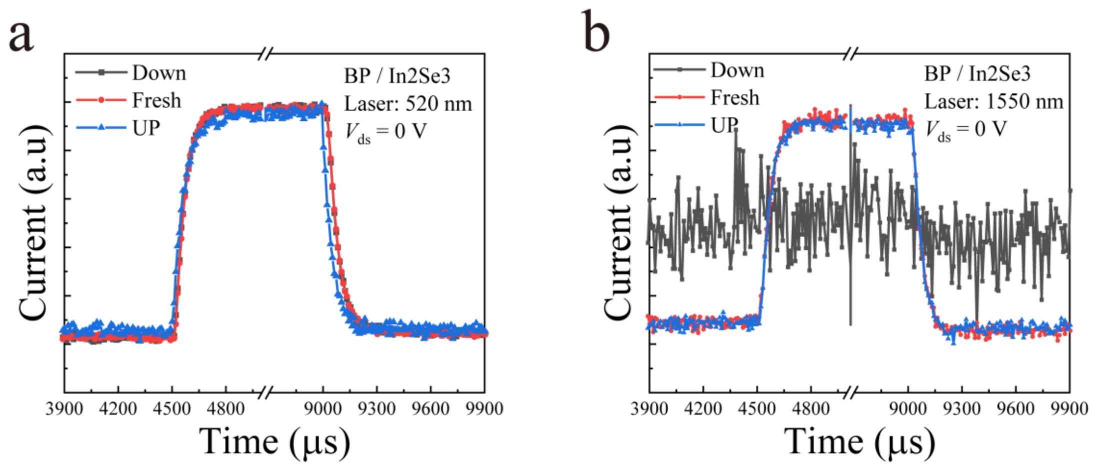

Supplementary Figure 12. Normalized I_{ph} under different ferroelectric polarization states at zero bias for (a) 520 nm and (b) 1550 nm incidence, respectively. Consistent response times were obtained for all ferroelectric polarization states.

Supplementary Table 1. Comparison of our heterojunction device with those reported works on the characteristic parameters.

Device	Work mechanism	Test condition	R (A/W)	Rising time(us)	Refs.
**p-GaN/α-In_2Se_3**	PV	V_{ds} =0V<br>V_g =0V	0.088 @365nm<br>0.07 @850nm	200000	[1]
**WSe_2/In_2Se_3**	PV	V_{ds} = -1V<br>V_g = -50V	26 @650nm	2220	[2]
**multilayer In_2Se_3**	PC	V_{ds} = 0.05V<br>V_g = 40V	98000 @640nm	9000000	[3]
**multilayer InSe**	PC	V_{ds} =3V<br>V_g = 0V	0.035 @632nm	5000	[4]
**MoS_2**	PC	-	0.00042 @550nm	50000	[5]
**α-In_2Se_3/3R MoS_2**	PV	V_{ds} =0.5V	2052 @ 520nm	20000	[6]
**In_2Se_3 monolayer**	PC	V_{ds} =2V	340@532	6000	[7]
**In_2Se_3 nanosheets**	PC	V_{ds} =5V	20.5@532nm	24600	[8]
**BP**	PC	V_{ds} =0.4V	0.135@1550nm	-	[9]
**BP**	PC	V_{ds} =0.2V	0.0048 @640nm	-	[10]
**BP**	PC	V_{ds} =-0.2V	0.02@520nm<br>0.05mA@1550nm	1000	[11]
**BP/α-In_2Se_3**	PV	V_{ds} =-0.5V<br>V_g =50V	2.88 @520nm<br>1.52 @1550 nm	-	This work
**BP/α-In_2Se_3**	PV	V_{ds} =0V<br>V_g =50V	0.16 @520nm<br>0.10 @1550 nm	176	This work

Supplementary Note 1. Band structure calculation of BP and In_2Se_3.

To confirm the band structure of BP and In_2Se_3, we calculate the Fermi level with the following formulas.

For nondegenerate semiconductor carriers that obey Boltzmann distribution $E_c - E_F \gg k_B T$ or $E_F - E_v \gg k_B T$

$$n_0 = N_c \exp\left[-\frac{(E_c - E_F)}{k_B T}\right] \tag{1}$$

$$p_0 = N_v \exp\left[-\frac{(E_F - E_v)}{k_B T}\right] \tag{2}$$

For degenerate semiconductor carriers that obey Fermi-Dirac distribution $E_c - E_F \leq k_B T$ or $E_F - E_v \leq k_B T$

$$n_0 = \frac{2}{\sqrt{\pi}} N_c F_{1/2}\left[-\frac{(E_c - E_F)}{k_B T}\right] \tag{3}$$

$$p_0 = \frac{2}{\sqrt{\pi}} N_v F_{1/2}\left[-\frac{(E_F - E_v)}{k_B T}\right] \tag{4}$$

where n_0/p_0 is the free electron/hole concentration at thermal equilibrium, $N_{c/v} = 2(\frac{2\pi m^*_{n/p} k_B T}{h^2})^{3/2}$ is the conduction/valence band equivalent density of states. $E_c - E_F$ and $E_F - E_v$ are the gaps from Fermi level to the minimum of conduction band and maximum of valence band, respectively. k_B is the Boltzmann constant, T is the temperature, $m^*_{n/p}$ is the electron/hole effective mass, and $F_{1/2}$ is the Fermi-Dirac integral function.

In order to precisely determine the Fermi energy levels, the mobility and carrier concentration of the materials also need to be obtained. Based on the measured pure material electrical data (Supplementary Figure 4) and $\mu = \frac{dI_{ds}}{dV_g} \cdot \frac{L}{W \cdot V_{ds} \cdot \varepsilon_0 \varepsilon_r / d}$, the mobilities of BP can be calculated as $201\, cm^2 V^{-1} s^{-1}$, where L and W are the channel length and width, ε_0 and ε_r are the dielectric constant of vacuum and SiO_2, and d is the thickness of the dielectric layer.

Then, the carrier concentration can be calculated by $n = \frac{\delta}{\mu q}$, where δ is the

conductivity in the linear region, q is the electron charge, and μ is the mobility calculated above. The volume concentration of BP with $p_0 = \frac{p}{h}$ is calculated as $1.48 \times 10^{18}\ cm^{-3}$. In the case of In_2Se_3, the volume concentration is 2.42×10^{16} cm^{-3} obtained with Hall test which is shown in Supplementary Figure 6c (In_2Se_3 is "Fresh" state). Here, $m^*_{h,BP} = 0.34\ m_0$.[12] For heavily doped BP, since the Fermi level is very close to the maximum of valence band, the holes are submitted to Fermi distribution. Therefore, we substituted p_0 into formula (4) to figure out $(E_\mathrm{F} - E_\mathrm{V})$ as 0.028 eV. For In_2Se_3, $(E_\mathrm{c} - E_\mathrm{F})$ is calculated to be 0.14 eV by the Equation (1).

Supplementary Note 2

We also calculate the BP/In_2Se_3 depletion zone distribution and concentrations of multilayer BP and In_2Se_3 under different ferroelectric polarization states with Supplementary Note1.
They are listed below:

		BP	In_2Se_3
Doping concentrations	P_{Down}	1.81×10^{19} cm^{-3}	3.60×10^{15} cm-^{3}
	P_{Fresh}	1.48×10^{18} cm^{-3}	2.40×10^{16} cm^{-3}
	P_{up}	8.69×10^{17} cm^{-3}	4.50×10^{17} cm^{-3}
Permittivity		8.3 ε0 [13]	17 ε0 [14]
Density of states		4.97×10^{18} cm^{-3}	1.02×10^{19} cm^{-3}

The depths d_1 and d_2 can be calculated through

$$d_1 = \left[\frac{2\varepsilon_1\varepsilon_2 N_{D2} V_D}{qN_{A1}(\varepsilon_1 N_{A1} + \varepsilon_2 N_{D2})}\right]^{1/2} \tag{5}$$

$$d_2 = \left[\frac{2\varepsilon_1\varepsilon_2 N_{A1} V_D}{qN_{D2}(\varepsilon_1 N_{A1} + \varepsilon_2 N_{D2})}\right]^{1/2} \tag{6}$$

The exact values are listed below:

		BP	In_2Se_3
depletion region	P_{Down}	0.10 nm	518.70 nm
	P_{Fresh}	3.07 nm	189.24 nm
	P_{up}	16.71 nm	32.26 nm

Supplementary Note 3

According to the reference 15, the open-circuit photovoltage V_{ph-oc} can be expressed as:

$$V_{ph-oc} = \frac{a\, K_b\, T}{q}\, Ln(\frac{I_{ph-sc}}{I_0} + 1) \qquad (7)$$

where a is the ideality factor, K_b is the Boltzmann constant, T is the absolute temperature, q is the elemental charge. Since I_{ph-sc} is almost linearly proportional to P_{in} (Figure 3(d)), Equation 7 can be modified as:

$$V_{ph-oc} = \frac{a\, K_b\, T}{q}\, Ln(\frac{P_{in}}{P_0} + 1) \qquad (8)$$

Here, P_{in} is the incident power. It indicates that the open-circuit voltage logarithmically increases with the incident power. The experimental data fits very well with Equation 8 as shown in Supplementary Figure 8.

References

[1] H. J. Jin, C. Park, K. J. Lee, G. H. Shin, S. Y. Choi, *Adv Mater Technol* **2021**, 2100494.

[2] B. Liu, B. Tang, F. Lv, Y. Zeng, J. Liao, S. Wang, Q. Chen, *Nanotechnology* **2020**, *31* (6), 065203.

[3] J. O. Island, S. I. Blanter, M. Buscema, H. S. van der Zant, A. Castellanos-Gomez, *Nano Lett* **2015**, *15* (12), 7853.

[4] L. Sidong, G. Liehui, N. Sina, G. Antony, K. Rajesh, L. Jun, C. Manish, Y. Hisato, G. Gautam, V. Robert, D. Aditya, Mohite., M. Pulickel, Ajayan., *ACS Nano* **2014**, *8* (2), 1263.

[5] Y. Zongyou, L. Hai, L. Hong, J. Lin, S. Yumeng, S. Yinghui, L. Gang, Z. Qing, C. Xiaodong, Z. Hua, *ACS Nano* **2011**, *6* (1), 74.

[6] W. Cai, J. Wang, Y. He, S. Liu, Q. Xiong, Z. Liu, Q. Zhang, *Nano-micro Lett* **2021**, *13* (1), 74.

[7] J. Zhou, Q. Zeng, D. Lv, L. Sun, L. Niu, W. Fu, F. Liu, Z. Shen, C. Jin, Z. Liu, *Nano Lett* **2015**, *15* (10), 6400.

[8] Z. Q. Zheng, J. D. Yao, G. W. Yang, *J Mater Chem C* **2016**, *4* (34), 8094.

[9] N. Youngblood, C. Chen, S. J. Koester, M. Li, *Nat Photonics* **2015**, *9* (4), 247.

[10] M. Buscema, D. J. Groenendijk, S. I. Blanter, G. A. Steele, H. S. J. van der Zant, A. Castellanos-Gomez, *Nano Lett* **2014**, *14* (6), 3347.

[11] M. Engel, M. Steiner, P. Avouris, *Nano Lett* **2014**, *14* (11), 6414.

[12] L. Li, G. J. Ye, V. Tran, R. Fei, G. Chen, H. Wang, J. Wang, K. Watanabe, T. Taniguchi, L. Yang, X. H. Chen, Y. Zhang, *Nat Nanotechnol* **2015**, *10* (7), 608.

[13] B. Deng, V. Tran, Y. Xie, H. Jiang, C. Li, Q. Guo, X. Wang, H. Tian, S. J. Koester, H. Wang, J. J. Cha, Q. Xia, L. Yang, F. Xia, *Nat Commun* **2017**, *8*, 14474.

[14] D. Wu, A. J. Pak, Y. Liu, Y. Zhou, X. Wu, Y. Zhu, M. Lin, Y. Han, Y. Ren, H. Peng, Y. H. Tsai, G. S. Hwang, K. Lai, *Nano Lett* **2015**, *15* (12), 8136.
